# Mangrove succession enriches the sediment microbial community in South China

**DOI:** 10.1038/srep27468

**Published:** 2016-06-06

**Authors:** Quan Chen, Qian Zhao, Jing Li, Shuguang Jian, Hai Ren

**Affiliations:** 1 Key Laboratory of Vegetation Restoration and Management of Degraded Ecosystems, South China Botanical Garden, Chinese Academy of Sciences, Guangzhou 510650, P. R. China; 2 University of Chinese Academy of Sciences, Beijing 100049, P. R. China

## Abstract

Sediment microorganisms help create and maintain mangrove ecosystems. Although the changes in vegetation during mangrove forest succession have been well studied, the changes in the sediment microbial community during mangrove succession are poorly understood. To investigate the changes in the sediment microbial community during succession of mangroves at Zhanjiang, South China, we used phospholipid fatty acid (PLFA) analysis and the following chronosequence from primary to climax community: unvegetated shoal; *Avicennia marina* community; *Aegiceras corniculatum* community; and *Bruguiera gymnorrhiza* + *Rhizophora stylosa* community. The PLFA concentrations of all sediment microbial groups (total microorganisms, fungi, gram-positive bacteria, gram-negative bacteria, and actinomycetes) increased significantly with each stage of mangrove succession. Microbial PLFA concentrations in the sediment were significantly lower in the wet season than in the dry season. Regression and ordination analyses indicated that the changes in the microbial community with mangrove succession were mainly associated with properties of the aboveground vegetation (mainly plant height) and the sediment (mainly sediment organic matter and total nitrogen). The changes in the sediment microbial community can probably be explained by increases in nutrients and microhabitat heterogeneity during mangrove succession.

Mangrove ecosystems are coastal wetlands dominated by woody plants that are adapted to saline, coastal soils. These ecosystems occur throughout the tropics and subtropics and are characterized by azonal plant communities that are greatly affected by ocean tides^1^,^2^. Because they are located in the ecotones of land–sea–estuary, mangrove ecosystems have high habitat heterogeneity. As a consequence, mangrove ecosystems are critical not only for sustaining global biodiversity but also for providing direct and indirect ecosystem services to humans^3^,^4^.

Like other forests, the mangrove forests at Zhanjiang, China exhibit different stages of succession^5^,^6^,^7^. At the beginning of the succession, the coast is a shoal without plants. The first mangrove to appear in this succession is usually *Avicennia marina*, which forms single-species communities. Then, *Aegiceras corniculatum* gradually appears in the tidal flat and forms an *A. marina* + *A. corniculatum* community or an *A. corniculatum* community (which sometimes includes a few *Kandelia candel* individuals). The above three communities are classified as the primary, early, and middle successional stage of mangroves. As sediments accumulate^8^, the habitat becomes more suitable for the following species that characterize the late successional stage: *Bruguiera gymnorrhiza*, *K. candel*, *Rhizophora stylosa*, and others. These species replace the previous communities and form a mixed, mature mangrove forest. The intertidal zone at Zhanjiang also supports an *Excoecaria agallocha* community (*E. agallocha* growing together with some other semi-mangrove species, such as *Hibiscus tiliaceus*, *Pluchea indica*, and *Clerodendrum inerme*), which can be considered transitional between a terrestrial community and an intertidal community. Although the changes in the aboveground vegetation during mangrove succession have been well studied, the changes in the belowground biota during mangrove succession are poorly understood.

Sediment microorganisms are important components of mangrove ecosystems^9^,^10^. They assist in the decomposition of organic matter and are critical for the cycling of nutrients and water^2^,^11^. Research has revealed a close relationship between soil microorganisms, nutrients, and plants in the recycling and conserving of nutrients in mangrove ecosystems^12^. The highly productive and diverse microbial community living in mangrove sediments continuously transforms nutrients bound in dead mangrove vegetation into nutrients that can be used by the living plants. In turn, root exudates serve as a food resource for the microorganisms. Soil microorganisms are also an essential food for protists and invertebrates, forming the base of benthic food webs and perhaps acting as a sink for carbon in estuaries^13^. Some plant growth-promoting rhizobacteria that aggressively colonize mangrove roots could be used in mangrove reforestation or restoration^14^. Finally, microorganisms often play an essential role in the bioremediation of polluted mangrove ecosystems^15^.

Because different plant communities support different microbial communities^16^,^17^, it is likely that microbial communities will change with succession in mangrove ecosystems^18^. This is in part because sediment characteristics differ among mangrove communities during succession. Research on the community structure of soil microorganisms in different mangrove successional stages should provide valuable information for the conservation and sustainable use of mangroves in South China.

In this paper, we describe the changes in the structure of the microbial community and in the environment during the succession of the mangrove forest at Zhanjiang, China. Using samples collected in the wet and dry season from four sites representing a mangrove chronosequence, we attempt to answer the following questions: 1) How does the sediment microbial community change with mangrove succession? and 2) What are the main factors that determine the structure of the sediment microbial community during mangrove succession?

## Results

### The sediment physicochemical properties in different successional stages of mangroves

The vegetation and sediment physiochemical properties of these mangrove communities were recently described and compared by Chen *et al.*^39^. In analyzing the data from Chen *et al.*, we found that sediment physicochemical properties were positively correlated with vegetation characteristics (Table 1). As the plant community matured and its complexity increased during mangrove succession, the accumulation of sediment biogenic elements (C, N, P and K) increased.

### The microbial community in different successional stages of mangroves

**Figure 1:**
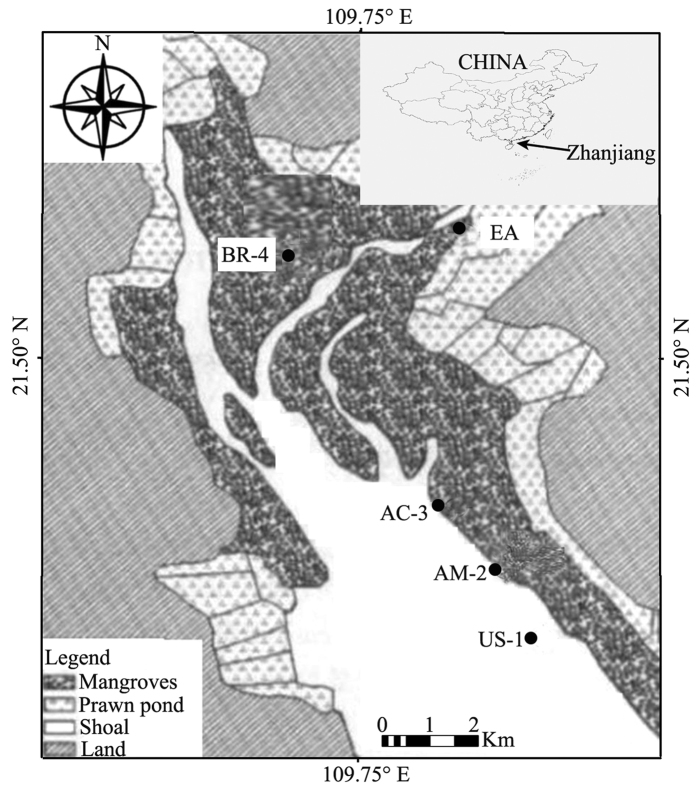
Locations of the four sites that represented a mangrove chronosequence at Zhanjiang, China. From primary to climax community, the sites were: US-1 = unvegetated shoal; AM-2 = *A. marina* community; AC-3 = *A. corniculatum* community; and BR-4 = *B. gymnorrhiza* + *R. stylosa* community. A fifth site (EA = *E. agallocha* community) was used for assessment of tidal effects (ArcGIS 10.2 http://www.esri.com/data/find-data).

**Figure 2:**
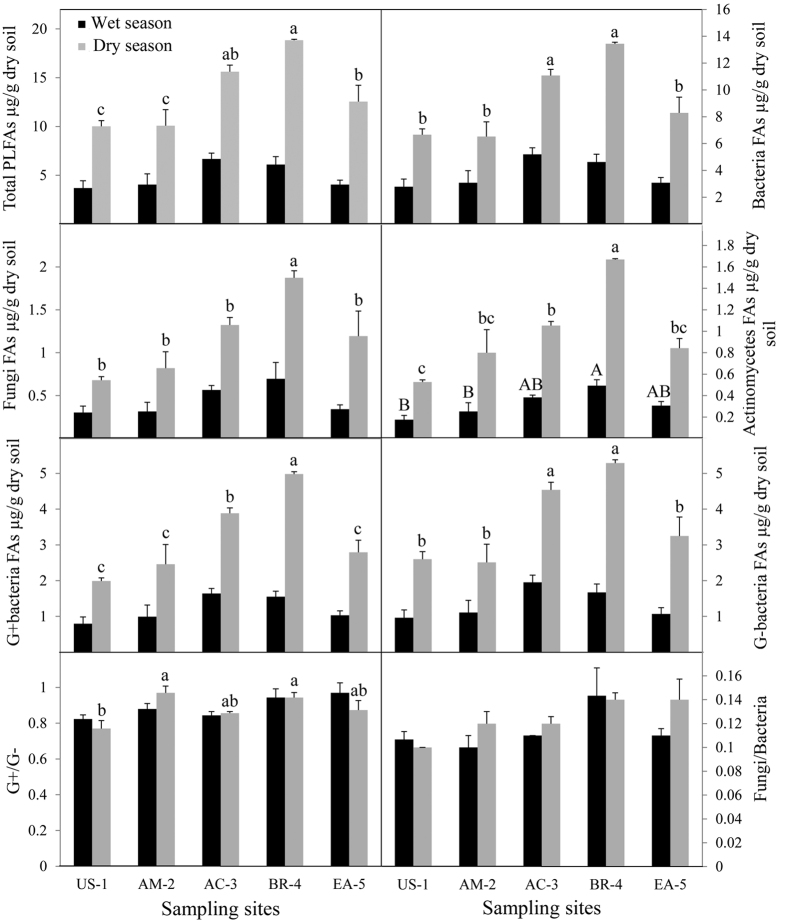
Concentrations of phospholipid fatty acids (PLFAs) of different sediment microbial groups at each sampling site at Zhanjiang, China. Uppercase and lowercase letters indicate significant differences in the wet season and dry season, respectively.

In both the wet and dry season, the abundances (as indicated by PLFA concentrations) of all microbial groups (total microorganisms, bacteria, fungi, gram-positive bacteria, gram-negative bacteria, and actinomycetes) and the ratios of fungi to bacteria and gram-positive bacteria to gram-negative bacteria increased with mangrove succession. In other words, the abundances or ratios were lowest in the unvegetated shoal (US-1), were intermediate in the *A. marina* community (AM-2) and *A. corniculatum* community (AC-3), and were highest in the *B. gymnorrhiza* + *R. stylosa* community (BR-4) (Figs 1 and 2). Values for all microbial groups in an *E. agallocha* community (EA-5, which was included for comparison of tidal effects) were often equal to those in AM-2 and AC-3. In both the wet and dry season, bacterial PLFAs accounted for most of the microbial PLFAs.

**Figure 3:**
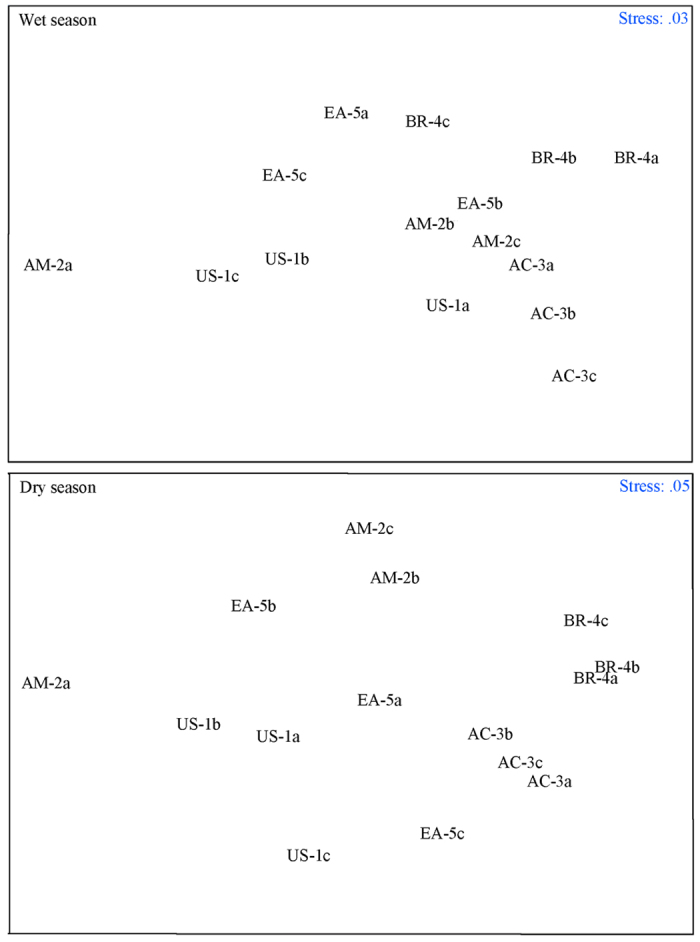
Non-metric multi-dimensional scaling (MDS) of the sediment microbial communities at the five sampling sites at Zhanjiang, China. (**a**,**b**,**c**) refer to the three plots at each site.

During the dry season, the abundances of total microorganisms, bacteria, fungi, actinomycetes, gram-positive bacteria, and gram-negative bacteria significantly differed (*p* < 0.05) among mangrove successional stages. During the wet season, the only microbial group whose PLFA concentrations significantly differed among the mangrove successional stages was the actinomycetes. According to non-metric multi-dimensional scaling (MDS), sediment microbial community structure differed among the mangrove successional stages (Fig. 3).

The abundances of total microorganisms, bacteria, fungi, actinomycetes, gram-positive bacteria, and gram-negative bacteria at all five sites were significantly higher in the dry season than in the wet season (Fig. 2). The ratios of fungi to bacteria and gram-positive bacteria to gram-negative bacteria were similar in the wet and dry season at all five sites. The interaction of season with site was significant (*p* < 0.05) for total microorganisms, bacteria, fungi, gram-positive bacteria, gram-negative bacteria, and actinomycetes (see Supplementary Information, Appendix S1).

### Environmental factors associated with the sediment microbial community

The abundances of total microorganisms, bacteria, fungi, gram-positive bacteria, and gram-negative bacteria were positively correlated with total sediment nitrogen (TN) in the wet season and with sediment organic matter (SOM) in the dry season (Table 2). Actinomycete abundance and the ratio of fungi to bacteria were positively correlated with SOM in the wet season and with plant height in the dry season.

**Figure 4:**
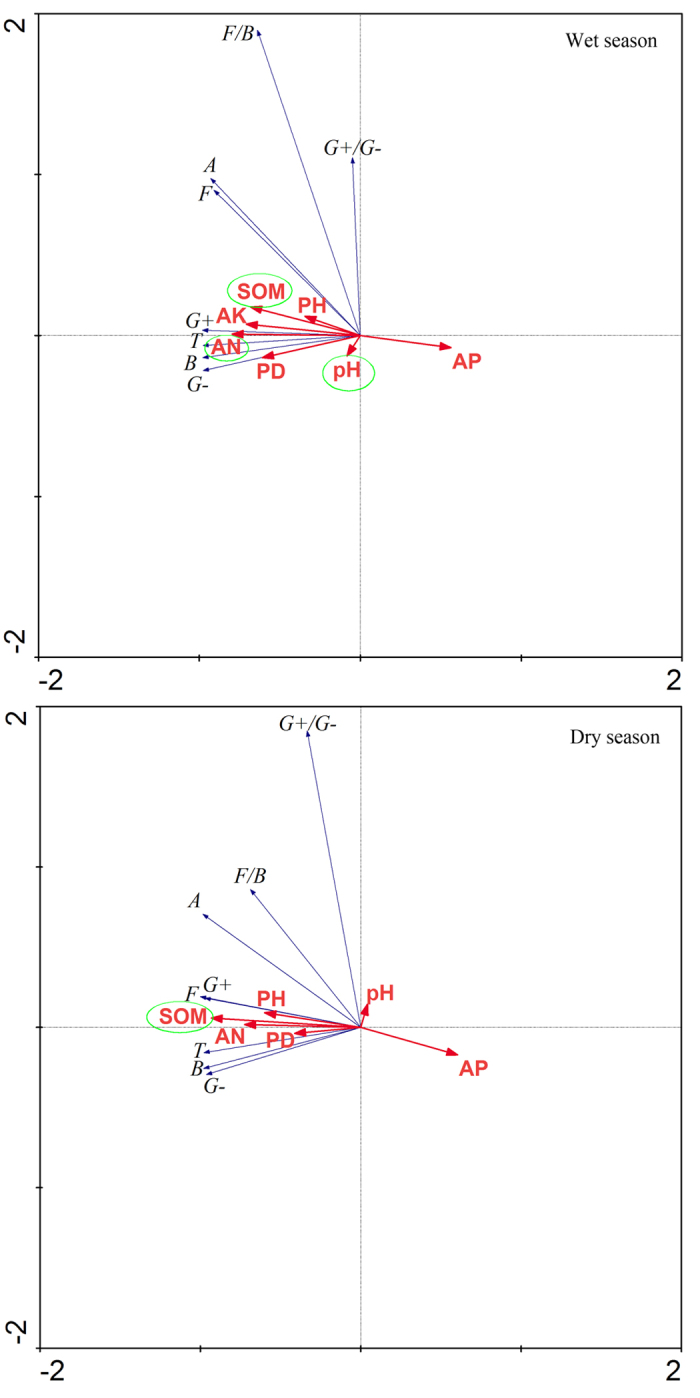
Biplot generated by canonical redundancy analysis (RDA) illustrating the effects of environmental factors on the PLFA concentrations of sediment microbial groups at the five sampling sites at Zhanjiang, China. Data for sediment and vegetation properties were from Chen *et al.*^39^ SOM = sediment organic matter; AN = sediment ammonium nitrogen; AP = sediment available phosphorus; AK = sediment available potassium; PD = plant density of mangrove; PH = plant height of mangrove. Abbreviations for PFLA concentrations: T = total microbial community; B = bacteria; F = fungi; A = actinomycetes; G+ = gram-positive bacteria; G− = gram-negative bacteria; G+/G− = ratio of gram-positive to gram-negative bacteria; F/B = ratio of fungi to bacteria.

According to canonical redundancy analysis (RDA), the main environmental variables associated with changes in the sediment microbial community during mangrove succession were sediment organic matter (SOM), sediment ammonium nitrogen (AN), sediment available phosphorus (AP), sediment available potassium (AK), and pH (Fig. 4). SOM, pH, AN, and AK explained 44, 23, 15, and 6%, respectively, of the total variation in the sediment microbial community in the wet season. SOM explained 83% of the total variation in the sediment microbial community in the dry season. All of the environmental variables identified by RDA were positively correlated with the abundances of the microbial groups.

## Discussion

Determining the mechanisms regulating the relationships between environmental factors and benthic organisms has been an active research area in estuarine ecology^19^,^20^. Macro-benthos in mangroves are controlled by a combination of factors, and no single factor can be considered as the main determinant^2^. Existing studies have indicated that different plant communities in mangrove forests result in changes in habitat that might affect the microbial community^21^,^22^. Although many reports have described the relationship between sediment physiochemical properties and the benthos community, few reports have considered the relationships between the characteristics of the mangrove plant community structure and the mangrove microbial community. Our results showed that both the sediment physicochemical properties and the aboveground vegetation properties affected the structure of the sediment microbial community during mangrove succession.

As mangrove succession advanced at Zhanjiang, the abundance of sediment microorganisms (as indicated by PLFA concentrations) increased remarkably (Fig. 2). Although previous research suggested that different mangrove communities might support different sediment microbial communities because of differences in litter inputs^2^,^23^, most previous studies focused on microbial species diversity and function or only a subset of the total microbial community^24^,^25^. For example, Wang *et al.* found that the changes in the community structure of ammonia- or ammonium-oxidizing microorganisms were associated with changes in leaf litter sources^26^. Bhattacharyya *et al.* reported that the distribution and diversity of archaeal taxa in mangrove sediment were greatly affected by the history of hydrocarbon/oil pollution^27^. Although these studies have provided valuable information, additional information, such as that provided in the current study, is needed about how the entire microbial community is affected by environment and by mangrove succession.

Microorganisms in mangrove sediments are likely to be greatly affected by nutrient availability. Most nutrients in such sediments are derived from litter decomposition and from mangrove secretions^28^. Previous research demonstrated that the microbial biomass in mangrove sediment is related to the contents of soil organic matter, total nitrogen, and available nitrogen^10^,^29^,^30^. During mangrove succession, the diversity and biomass of the plant community increase; as a consequence, the mature mangrove community provides an abundance of sediment biogenic matter, such as SOM, TC, TN, TP and TK (Table 1), all of which result in an increase in available substrates for microorganisms^8^,^7^,^31^.

The structural characteristics of the mangrove forest during succession also affect the benthic microbial community. Increases in plant height and crown breadth enhance the complexity of rhizome structure and structural heterogeneity of the surface environment^32^. This structural heterogeneity in turn greatly increases the complexity of the epibenthic and shallow endobenthic environment and enriches the microhabitats for benthic microorganisms^2^,^31^. Furthermore, the late successional stage (or mature) mangrove communities usually have high tidal levels, short durations of tidal inundation, reduced sediment erosion, and low sediment salinity, which increase the suitability of the sediment as a microbial habitat^29^.

Protozoa are important predators of bacteria and undoubtedly affect bacterial biomass^33^. An increase in protozoa can result in an increase in predation of bacteria and a reduction in bacterial biomass^34^. We found that the abundance of benthic protozoa decreased with mangrove succession in the same area (see Supplementary Information, Appendix S2). Consequently, a decrease in protozoan predation may partly explain the increase in microbial biomass with mangrove succession.

In our study, PLFA concentrations of microbial groups were significantly higher in the dry season than in the wet season. This may be due in part to litter input^35^,^36^, which is greater in the dry season at Zhanjiang. Zhang found that temperature is the main factor associated with the seasonal change in the microbial community in mangrove sediments^29^. Additionally, plants and microorganisms can compete for nutrients^37^, and mangroves may compete strongly in the wet season when temperature and moisture result in vigorous plant growth and thus an increased demand for nutrients. During the dry season, in contrast, low temperatures and arid conditions reduce the mangrove growth rate and therefore reduce the demand and competition for nutrients.

In summary, microbial abundance (as indicated by PLFA analysis) was lowest in the unvegetated shoal perhaps because the unvegetated shoal provided an inadequate food supply (sediment organic matter and total nitrogen), low habitat heterogeneity (plant height), and high predation pressure (protozoa abundance). Microbial abundance was highest in the most mature mangrove forest perhaps because the mature forest provided an adequate food supply, substantial habitat heterogeneity, and reduced predation. Changes in the microbial community in sediment with mangrove succession were mainly associated with changes in nutrient quantity and microhabitat heterogeneity. These results increase our understanding of the biodiversity in mangroves and should help guide the conservation and sustainable use of this and perhaps other mangrove forests.

## Methods

### Site description

A field study was conducted at the Zhanjiang Mangrove National Nature Reserve (109°40′–110^°^35′ E, 20°14′–21^°^35′ N), which is located along the coastal shoal of the Leizhou Peninsula, Guangdong, China. The reserve is in a transitional region between north tropical and subtropical climates. The mean annual temperature is 23.8 °C, the mean coldest monthly temperature is 17.2 °C, and the seawater surface mean temperature is 23.7 °C. There is no frost during the year. The mean annual precipitation is 1800 mm, and most precipitation occurs during the summer rainy season or monsoon. The intertidal zone is characterized by one or two tidal cycles per day. The tidal range is approximately 2 m. In 2002, this reserve was listed among the internationally important wetlands by the Ramsar Convention; it is especially important as a habitat for waterbirds.

Because mangrove forests require a long time (usually over 100 years) to complete the succession from unvegetated shoal to mature, mixed-mangrove forest, we selected four sites as a chronosequence. Each site supported a mangrove community at a different stage of succession as described by Chen *et al.*^39^, who studied the macrobenthic faunal communities at these sites. Site US-1 was an unvegetated shoal (the primary successional stage). Site AM-2 supported an *A. marina* community (the early successional stage). Site AC-3 supported an *A. corniculatum* community (the middle successional stage), and site BR-4 supported a community dominated by *B. gymnorrhiza* + *R. stylosa* (the late successional stage). An additional nearby site, EA, supported an *E. agallocha* community, which was used for comparative purposes to show the effects of different tidal regimes on the sediment microbial community. Sites US-1, AM-2, AC-3, and BR-4 were located in the low-tide zone and were periodically inundated by the tide. Site EA was located in the high-tide zone near the backshore and was not periodically inundated by the tide. The five sites were located in the northwest part of the reserve (Fig. 1).

### Field measurements

On 18 July 2011 (during the wet season) and on 26 December 2011 (during the dry season), three replicate plots (10 m × 10 m) at each site were designated for sediment sampling. The plots, which were separated by at least 100 m at each site, were located at an equal distance from the high tidal mark at each site and were inundated and exposed with the daily tidal cycle. Samples were collected with a shovel at low tide, when the plots were not inundated. The raw data for vegetation and sediment physiochemical properties obtained by Chen *et al.*^39^ were used to analyze the correlation between these properties and microbial properties in the current study, as described later in this paper.

### Laboratory analyses

PLFA analysis was used to determine the composition of the microbial community and was performed as described by Bossio & Scow^35^ and Abaye *et al.*^40^. The extracted fatty acid methyl esters (FAMEs) were separated, quantified, and identified using capillary gas chromatography (GC) with an Agilent 6890 gas chromatograph (Agilent Technologies, Palo Alto, CA, USA) and the MIDI Sherlock Microbial Identification System (MIDI Inc., Newark, DE, USA). A non-polar column (95% dimethyl, 5% diphenyl polysiloxane, 30 mm long × 0.25 mm internal diameter, film thickness 320 μm) was used to separate the PLFAs. The oven temperature was kept at 70 °C for 1 minute, was then increased to 150 °C at 5 °C /minute, and was then further increased to 280 °C at 5 °C /minute, before it was held at 280 °C for 5 minutes. Fatty acids were quantified by calibration against standard solutions of FAME 19:0 (Matreya Inc., State College, PA, USA), which was added as an internal standard at a concentration of 50 ng/ml.

The fatty acids used as biomarkers for specific groups of soil microorganisms are listed in Table 3. The quantities (ng/g dry soil) of specific fatty acids in a given sample were determined with the following formula^40^:


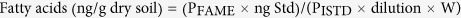


where PFAME is the peak area of each fatty or acid methyl ester, PISTD is the peak area of the internal standard, ng Std is the concentration of the internal standard (ng/μl solvent), and W is the oven-dry soil weight. We assumed that PLFA concentrations for microbial groups were indicators of group abundances.

### Statistical analyses

One-way analysis of variance (ANOVA) was used to compare microbial communities among the five sites. Two-way ANOVAs were used to compare microbial communities among sites and seasons. The similarity of the sediment microbial communities among the sites was determined using the Bray-Curtis similarity coefficient for non-metric multi-dimensional scaling (MDS). Regression and canonical redundancy analysis (RDA) were used to investigate the relationships between environmental factors (sediment physicochemical properties and vegetation characteristics of mangrove communities; as previously noted, these data were obtained from Chen *et al.*)^39^ and the microbial communities at the different sites and in different seasons. Significance was set at *p* ≤ 0.05. The ANOVAs and regression analyses were performed using SPSS (SPSS ver. 20, IBM). MDS were performed with PRIMER software (Plymouth Routines in Multivariate Ecological Research ver. 7.0). RDA was performed with Canoco for Windows 5.0.

## Additional Information

**How to cite this article**: Chen, Q. *et al.* Mangrove succession enriches the sediment microbial community in South China. *Sci. Rep.* **6**, 27468; doi: 10.1038/srep27468 (2016).

## Supplementary Material

Supplementary Information

## Figures and Tables

**Table 1:** Relationships between sediment physicochemical properties and vegetation characteristics in a mangrove chronosequence at Zhanjiang, China as indicated by stepwise regression analyses.

	Sediment physicochemical properties	Vegetation characteristics	*P*	r^2^	Regression equation
Wet season	SOM	CB, PD	0.007	0.787	SOM = 0.685 + 0.362*CB + 0.948*PD
	TN	PD, CB	0.001	0.769	TN = 0.054 + 0.062*PD + 0.011*CB
	TP	PD	0.002	0.518	TP = 0.036 + 0.027*PD
	TK	PD	0.001	0.571	TK = 0.596 + 0.377*PD
	AP	CC, PH	0.010	0.919	AP = 14.842-8.322*CC-2.383*PH
	AN	PD	0.003	0.494	AN = 9.090 + 6.837*PD
	AK	–	–	–	–
	Salinity	–	–	–	–
Dry season	SOM	CB, PD	0.007	0.815	SOM = 0.804 + 0.364*CB + 0.854*PD
	TN	CB, PD	0.002	0.740	TN = 0.050 + 0.012*CB + 0.050*PD
	TP	PD	0.004	0.479	TP = 0.039 + 0.023*PD
	TK	PD	0.002	0.532	TK = 0.664 + 0.401*PD
	AP	CC	<0.001	0.897	AP = 23.859-21.966*CC
	AN	PD	0.001	0.554	AN = 4.176 + 3.276*PD
	AK	PD	0.001	0.600	AK = 458.705 + 404.695*PD
	Salinity	PD	0.001	0.576	Salinity = 17.512 + 6.569*PD

The data were collected by Chen *et al.* 39, and the analysis was conducted as part of the current study. SOM = sediment organic matter content; TN = total sediment nitrogen content; TP = total sediment phosphorus content; TK = total sediment potassium content; AP = sediment available phosphorus content; AK = sediment available potassium content; AN = sediment ammonium nitrogen content.

PH = plant height; CB = plant crown breadth; CC = coverage/canopy density; PD = plant density.

**Table 2:** Relationships between sediment microbial phospholipid fatty acids (PLFAs) and sediment/vegetation properties in the wet and dry season in a mangrove chronosequence at Zhanjiang, South China as indicated by stepwise regression analyses.

Microbial group	Wet Season			Dry Season		
	Sediment and vegetation properties	*p*	r^2^	Sediment and vegetation properties	*p*	r^2^
Total PLFAs	+TN	0.013	0.905	+SOM	0.004	0.955
Bacteria	+TN	0.017	0.885	+SOM	0.007	0.939
Fungi	+TN	0.005	0.950	+SOM	0.001	0.981
Actinomycete	+SOM	0.003	0.960	+PH	0.015	0.897
G+bacteria	+TN	0.008	0.932	+SOM	0.002	0.976
G−bacteria	+TN	0.023	0.862	+SOM	0.012	0.909
G+/G−	ns	–	–	ns	–	–
Fungi/Bacteria	+SOM	0.038	0.807	+PH	0.015	0.897

“+” = Positive correlation; ns = nonsignificant correlation; TN = total sediment nitrogen; SOM = sediment organic matter; PH = plant height of mangrove. Data for sediment and vegetation properties were from Chen *et al.*^39^

**Table 3:** PFLA biomarkers used to identify specific groups of sediment microorganisms at Zhanjiang, China.

Group or ratio	Biomarkers
General bacteria	*i14:0, 14:0, 15:0, i15:0, a15:0, 16:0, i16:0, 16:1w7c, 16:1w9c, 16:12OH, 17:0, i17:0, a17:0, cy17:0, 18:0, 18:1w7c, cy19:0, 10Me16:0, 10Me17:0, cy19:0w8c, 19:0, i19:0*
Gram-positive bacteria	*i13:0, i14:0, i15:0, a15:0, i16:0, i17:0, a17:0, i18:0, 10Me16:0*
Gram-negative bacteria	*15:0 3OH, 15:1w6c, 16:12OH, 16:1w5 7 9c, 17:1w8c, 18:1w7 9c, cy17:0*
Fungi	*18:2w6 9c, 20:1w9c, 18:1w9c, 16:1w5c*
Actinomycetes	*10Me 16:0, 10Me 17:0, TBSA 10Me18:0*
Gram-positive : Gram-negative	(*i13:0, i14:0, i15:0, a15:0, i16:0, i17:0, a17:0, i18:0, 10Me16:0*)*/*(*15:0 3OH, 15:1w6c, 16:12OH, 16:1w5 7 9c, 17:1w8c, 18:1w7 9c, cy17:0*)
Fungi : Bacteria	(*18:2w6 9c, 20:1w9c, 18:1w9c, 16:1w5c*)*/*(*i14:0, 14:0, 15:0, i15:0, a15:0, 16:0, i16:0, 16:1w7c, 16:1w9c, 16:12OH, 17:0, i17:0, a17:0, cy17:0, 18:0, 18:1w7c, cy19:0, 10Me16:0, 10Me17:0, cy19:0w8c, 19:0, i19:0*)

The prefixes *a*, *i*, and *cy* refer to anteiso, iso, and cyclopropyl branching, respectively; *br* indicates that the type of branching is unknown, while a number followed by Me indicates the position of the methyl group. Numbers preceded by *w* indicate the position of OH groups from the aliphatic end of the fatty acids^35^,^41^,^42^,^43^,^44^,^45^,^46^.
