# Supplementary material for: Mangrove succession enriches the sediment microbial community in South China
Source: Sci Rep. 2016 Jun 6;6:27468. doi: 10.1038/srep27468 (PMC4893734; doi:10.1038/srep27468)

**Mangrove succession enriches the sediment microbial community in South China**

Quan Chen^1,2^, Qian Zhao^1,2^, Jing Li^1^, Shuguang Jian^1*^, Hai Ren^1*^

^1^Key Laboratory of Vegetation Restoration and Management of Degraded Ecosystems, South China Botanical Garden, Chinese Academy of Sciences, Guangzhou 510650, P.R. China

^2^University of Chinese Academy of Sciences, Beijing 100049, P.R. China

*Corresponding. E-mail: [jiansg@scbg.ac.cn](mailto:jiansg@scbg.ac.cn), renhai@scbg.ac.cn

**Appendix S1**

The results of two-way analysis of variances (ANOVAs).

| Source | | Type III Sum of Squares | df | Mean Square | F | Sig. |
| --- | --- | --- | --- | --- | --- | --- |
| Season * Sampling sites | Total PLFAs | 43.601 | 4 | 10.900 | 3.917 | .017 |
|  | Bacteria | 27.198 | 4 | 6.799 | 4.707 | .008 |
|  | G-positove | 4.640 | 4 | 1.160 | 5.952 | .003 |
|  | G-negative | 4.623 | 4 | 1.156 | 4.147 | .013 |
|  | Fungi | .586 | 4 | .147 | 2.484 | .077 |
|  | G+/G- | .030 | 4 | .008 | 1.715 | .186 |
|  | Actinomycetes | .587 | 4 | .147 | 7.076 | .001 |
|  | Fungi/Bacteria | .001 | 4 | .000 | 1.005 | .428 |

*p* = Sig.

**Appendix S2**

Abundance, diversity, and richness of the benthic protozoan community at the five sampling sites representing a mangrove succession at Zhanjiang, China. Sediment samples were collected in the wet and dry season. US-1 = unvegetated shoal. AM-2 = *A. marina* community. AC-3 = *A. corniculatum* community. BR-4 = *B. gymnorrhiza* + *R. stylosa* community. EA = *E. agallocha* community. Values are means + SE.


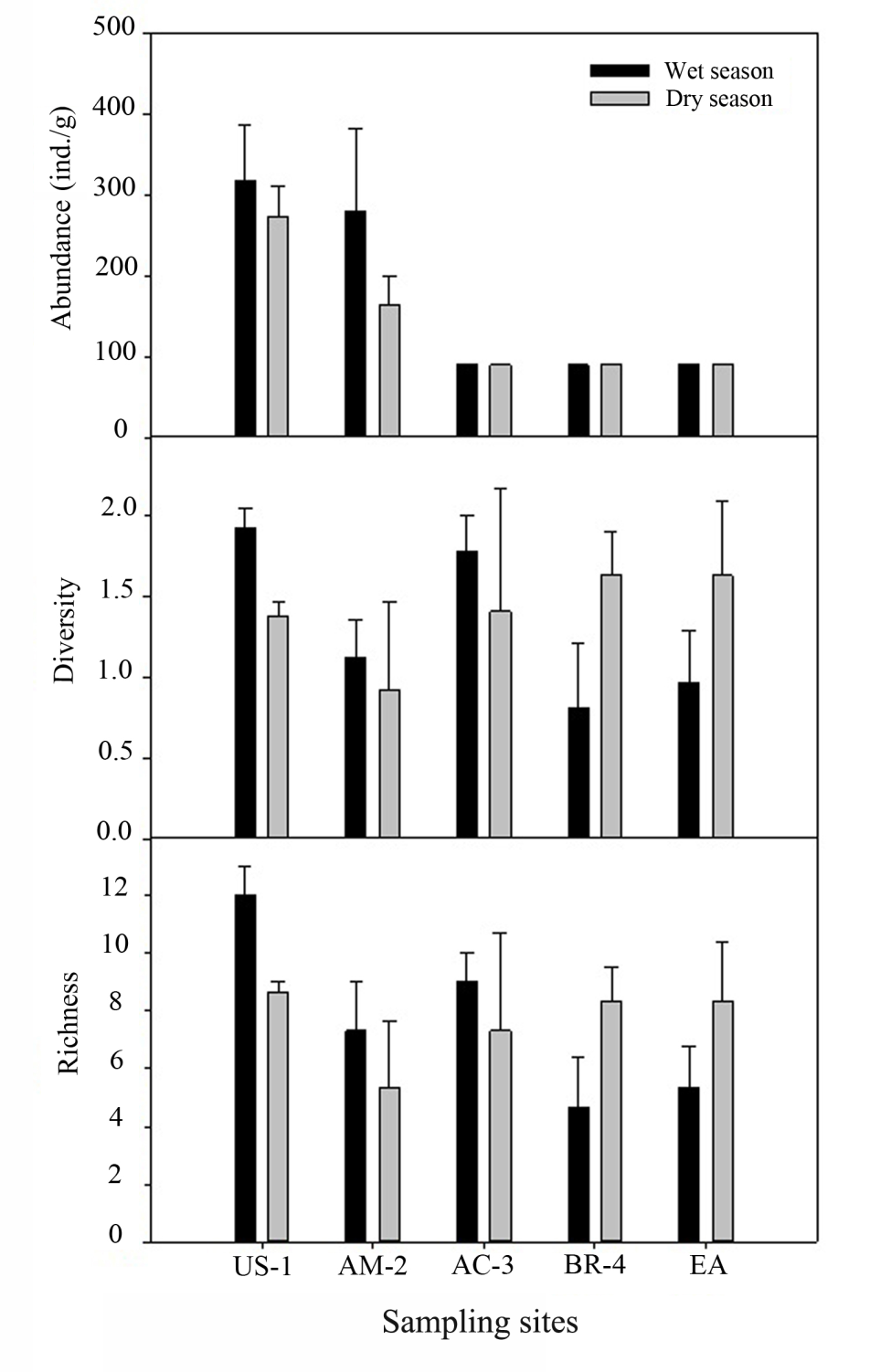

Supplement: Supplementary Information [file srep27468-s1.doc]
